# Is logistically motivated *ex vivo* lung perfusion a good idea?

**DOI:** 10.3389/frtra.2022.988950

**Published:** 2022-12-14

**Authors:** Caroline Van De Wauwer, Vincent van Suylen, Zhang L. Zhang, Erik A. M. Verschuuren, Wim van der Bij, C. Tji Gan, Rinse Ubbink, Michiel E. Erasmus

**Affiliations:** ^1^Department of Cardiothoracic Surgery, University of Groningen, University Medical Centre Groningen, Groningen, Netherlands; ^2^Department of Pulmonary Diseases and Lung Transplantation, University of Groningen, University Medical Centre Groningen, Groningen, Netherlands

**Keywords:** *ex vivo* lung perfusion, lung transplantation, primary graft dysfunction, logistic, medical

## Abstract

*Ex vivo* lung perfusion (EVLP) is a technique for reconditioning and evaluating lungs. However, the use of EVLP for logistical reasons is still under discussion. In this retrospective study, all EVLPs performed between July 2012 and October 2019 were analyzed for ventilation and perfusion data. After transplantation, primary graft dysfunction (PGD), lung function, chronic lung allograft dysfunction (CLAD)-free survival, and overall survival were analyzed. Fifty EVLPs were performed: seventeen logistic EVLPs led to 15 lung transplantations (LT) and two rejections (LR), and 33 medical EVLPs resulted in 26 lung transplantations (MT) and seven rejections (MR). Pre-EVLP PaO_2_ was lower for MT than LT (*p* < 0.05). Dynamic lung compliance remained stable in MT and LT but decreased in MR and LR. Plateau airway pressure started at a higher level in MR (*p* < 0.05 MT vs. MR at T60) and increased further in LR. After transplantation, there were no differences between MT and LT in PGD, lung function, CLAD-free survival, and overall survival. In addition, the LT group was compared with a cohort group receiving standard donor lungs without EVLP (LTx). There were no significant differences between LT and LTx for PGD, CLAD-free survival, and overall survival. FVC was significantly lower in LT than in LTx after 1 year (*p* = 0.005). We found that LT lungs appear to perform better than MT lungs on EVLP. In turn, the outcome in the LT group was comparable with the LTx group. Overall, lung transplantation after EVLP for logistic reasons is safe and makes transplantation timing controllable.

## Introduction

Since the first clinically successful lung transplantation (LTx) in 1983, a shortage of suitable donor lungs has been the main obstacle to helping patients with irreversible end-stage pulmonary disease. Only 15–25% of the organs can be used for transplantation, resulting in increased mortality in the group of individuals on the waiting list (1, 2).

The introduction of *ex vivo* lung perfusion (EVLP) in 2001 by Stig Steen (3), and further modification and fine-tuning of the technique by Cypel et al. (4), made it possible to assess and recondition marginal donor lungs that may be suitable for transplantation in a controlled environment. This resulted in a conversion rate of 34–100% (5).

Furthermore, EVLP makes it possible to go beyond the current narrow window of 6–8 h of cold preservation, extending preservation time (6–8) and enabling elective daytime lung transplantation (9).

Failure to identify inferior-quality standard donor lungs can result in severe primary graft dysfunction (PGD) after transplantation, which is associated with chronic lung allograft dysfunction (CLAD). EVLP has proven to be a unique and safe technique for identifying and excluding donor lungs with an increased risk of development of EVLP (8).

In July 2012, EVLP was introduced in our center to assess marginal donor lungs that did not fulfill the acceptance criteria. In the last 2 years, we also used EVLP to bridge the night-time period, making elective daytime lung transplantation possible.

In this study, we investigated whether logistic EVLP of lungs already determined suitable for transplantation is safe and has ventilation and perfusion characteristics during EVLP that are comparable with medically indicated EVLP. In addition, PGD, CLAD-free survival, and overall survival were analyzed for logistic and medical EVLP. Furthermore, the logistic EVLP group was compared with a cohort group that received standard donor lungs without EVLP.

## Materials and methods

### Study groups

Between July 2012 and October 2019, 50 EVLPs were performed in a single center for either medical or logistical reasons. For this retrospective study, data from EVLP and 41 lung transplant recipients were analyzed. Secondly, propensity score matching was performed in order to match baseline recipient and donor characteristics between the group receiving a standard lung transplantation and the group receiving a lung transplantation after EVLP for logistical indication. This study was approved by the institutional review board (Approval Number, METc 2021/339; UMCG research Register Number, 202100305).

### Donors and recipients

Donor lungs were offered through Eurotransplant. The inclusion criteria for medical EVLP were lungs (1) with a partial arterial oxygen pressure/fraction of inspired oxygen ratio (PaO_2_/FiO_2_) < 40 kPa at a positive end-expiratory pressure (PEEP) of 5 cmH_2_O and 100% oxygen, with clinically evident lung edema, (2) with a persistently low PaO_2_/FiO_2_ after active lung recruitment, (3) with persistent atelectasis after recruitment, (4) deemed to be of questionable quality during the organ donation procedure, and (5) that met extended DCD donor criteria. Exclusion criteria were as follows: (1) pulmonary hypertension in the donor; (2) drowning; (3) proven aspiration; (4) bilateral purulent pneumonia; and (5) bilateral lung contusion. The criteria followed for EVLP are described in the national EVLP protocol (Medisch protocol *ex vivo* long perfusie Nederland).

Logistic EVLP was started when donor lungs arrived after midnight in the transplant center, or when there were additional circumstances that made EVLP necessary (e.g., heart/lung offer from the same donor or from a different donor at the same time, or a combined lung-liver transplantation).

Recipient selection and donor/recipient matching were performed using international guidelines. Allocation was based on the lung allocation score (LAS).

### EVLP

EVLP was performed using our EVLP protocol described previously (10). The duration of medical EVLP was between 4 and 6 h, and of logistical EVLP was between 3 and 6 h. Medical EVLP was abandoned earlier if the lungs deteriorated beyond repair during the first hours of EVLP. In both situations, the accepted first cold ischemia time and the second cold ischemia time were at least 1 h to enable adequate cooling of the lungs.

### *Ex vivo* lung function evaluation

Graft assessment was undertaken hourly during EVLP. The perfusate was deoxygenated 10 min before evaluation. Simultaneously, the FiO_2_ was set at 100% and the respiratory frequency was increased from 7 to 10/minute. Tidal volume was set at 10 ml/kg for the first 40 EVLPs. In later EVLPs, the tidal volume was kept at 7 ml/kg.

Acceptance or decline of the lungs was based on the institutional and national protocol for EVLP.

### Baseline characteristics

Data from the resulting 41 LTx and the 30 propensity score-matched standard LTx were reviewed for donor and recipient variables. Age, gender, donor type, height, total lung capacity (TLC), body mass index (BMI), most recent pre-donation PaO_2_, and cause of death were recorded as donor variables.

For the EVLP cases, flush time, first ischemic time (cold ischemia from pulmoplegia in the donor up to the start of the EVLP [1^st^ CIT]), second ischemic times (cold ischemia from cooling during EVLP up to the reperfusion of the first implanted lung [2^nd^ CIT 1° lung] and up to the reperfusion of the second implanted lung [2^nd^ CIT 2^nd^ lung]), EVLP duration, and EVLP conversion rate were recorded as preservation variables. In the standard LTx group, cold ischemia from pulmoplegia in the donor up to the start of reperfusion of the first implanted lung (CIT1) and second implanted lung (CIT2) was recorded.

Recipient variables collected were age, gender, BMI, lung disease, LAS, single or bilateral LTx, use of cardiopulmonary bypass or extracorporeal life support, last FEV_1_ and FVC as a percentage of their predicted value before transplantation, ICU stay, and hospital stay.

### Endpoints

PGD, lung function, CLAD-free survival, and overall survival were the primary endpoints. PGD was graded in accordance with the ISHLT PGD definition and assessed at 48 and 72 h after LTx (T48 and T72) (11). Lung function, FEV_1_, and FVC, were assessed at 3 and 6 months, and after 1 year at the outpatient clinic. FEV_1_ and FVC are expressed as a percentage of the predicted value. CLAD was defined as a persistent decline in FEV_1_ (< 80% of baseline) (12). The secondary endpoint was pulmonary graft function during EVLP.

### Statistical analysis

An unpaired *t*-test was used to determine significant differences in pulmonary graft function and lung function. For within-group differences, a one-way ANOVA with a Bonferroni multiple comparison test was used. Data regarding pulmonary graft function and lung function are expressed as mean ± standard error of the mean. All other data are expressed as median ± range, unless stated otherwise.

Mann–Whitney *U*-tests were performed to compare non-normally distributed data. For nominal variables, either a χ^2^ test or Fisher's exact test was used; these variables are expressed in percentages and numbers. Overall survival and CLAD-free survival were visualized using the Kaplan–Meier method. Log-rank tests were performed to compare survival distributions. *P* < 0.05 was considered significant. All calculations were performed using IBM SPSS Statistics 25.0 software (IBM Corp., Armonk, NY, USA).

For propensity score matching, a multiple regression analysis was performed with variables that theoretically may have biased treatment (lung transplantation after logistical EVLP vs. standard lung transplantation without EVLP). Moreover, all baseline donor and recipient variables that differed between the two groups in univariate analyses (*p* < 0.10) were included. The EVLP group (*n* = 15) was matched to the standard lung transplantation without EVLP group (*n* = 30) in terms of donor age, donor gender, donor PaO_2_, donor smoking history, donor type (donation after circulatory death [DCD] or donation after brain death [DBD]), recipient gender, recipient age, recipient underlying lung disease, diabetes mellitus, preoperative mechanical ventilation, preoperative ECLS, and lung allocation score (LAS). For donation after euthanasia cases, the paO_2_ was defined as 60 kPa, as no arterial blood gas analyses are performed in these cases.

## Results

In total, 50 EVLPs were performed, of which 17 were logistically motivated EVLPs, resulting in 15 lung transplantations (LT) and two rejections (LR). Evident pulmonary edema was the reason for the rejections. Pathological examination of the rejected lungs showed hyperinflation (*n* = 1) and diffuse alveolar damage (*n* = 1).

The other 33 EVLPs were performed for medical reasons, resulting in 26 lung transplantations (MT) and seven rejections (MR). Indications for EVLP and reasons for rejection in the medically motivated group are listed in Table 1. In the declined cases, pathological examination revealed pulmonary edema (*n* = 1), bilateral bronchopneumonia with an abscess in the left lower lobe (*n* = 1), acute pneumonia (*n* = 1), and no abnormalities (*n* = 1). In three cases, the lungs were not sent for pathology.

**Table 1:** Indications for EVLP and reasons for rejection in the medical EVLP group.

**Indications for medical EVLP**
Low PaO_2_ (*n* = 14)
Combination of low PaO_2_ and atelectasis (*n* = 7)
Edema (*n* = 5)
Persistent atelectasis (*n* = 4)
Pulmonary embolism (*n* = 2)
Miscellaneous (*n* = 1)
**Reasons for rejection after medical EVLP**
Lung edema (*n* = 2)
Infection/edema in one lung (*n* = 3; in two cases there was no recipient available for the other lung)
Impossible to ventilate the lung (*n* = 1)
Technical failure (*n* = 1)

Five out of 50 lungs were changed from a supine position to a prone position during EVLP. Atelectasis of the dorsal part of the lungs was the reason for the prone position in two EVLPs for logistical reasons. The change of position improved the ventilation of the lungs. In one EVLP for logistical reasons, the prone position failed to prevent the development of pulmonary edema. In two EVLPs for medical reasons, the lungs were turned to a prone position in the absence of pO_2_ improvement. This resulted in better oxygenation and ventilation, and acceptance for transplantation.

The duration of the EVLP in MR was between 75 and 300 min. In LR, the EVLP was stopped after 210 and 215 min.

Thirty-eight donation procedures were planned during the evening/night (16/17 in the L group and 22/33 in the M group). The 16 EVLPs for logistical reasons were planned so that the transplantation could start during the day. In three logistical cases, additional circumstances made EVLP necessary. In one case, the heart and lungs were obtained from the same donor. As only one team was available during the night, the lungs were preserved during the EVLP. One patient received a combined lung-liver transplant. The liver transplantation was performed first, during which the lungs were preserved during the EVLP. In another case, we accepted heart and lungs from a different donor. During the holidays, there is less theater availability. Therefore, the lungs were preserved during the EVLP. This made it possible to perform the elective cardiac surgery planned for that day and both transplantations. In the MT group, EVLP was started immediately when the lungs arrived at the hospital. From the total of 38, 12 transplantations were performed during the evening/night and 14 were performed during the day.

The 15 logistical EVLPs that resulted in lung transplantation were matched with a group of 30 patients that received standard donor lungs without EVLP. In the conventional LTx group, CIT1 was 338 min (301–375 min) and CIT2 was 457 min (401–498 min) (**Table 5**).

In the LT group, the first CIT was 257 min (232–302), the second CIT of the first implanted lung was 237 min (128–266) and of 363 min (268–398) for the second implanted lung.

### Donor characteristics

Donor characteristics are shown in Tables 2, 3. Most recent pre-donation PaO_2_ at 100% oxygen was significantly different between the medical EVLP group and the logistical EVLP group. None of the other variables differed (Table 2).

**Table 2:** Donor characteristics of medical EVLP vs. logistical EVLP (non-transplanted cases included).

	***n* (a)**	***M* (*n* = 33)**	***n* (a)**	***L* (*n* = 17)**	***p*-value**
Age (years)	33	47.0 (37.5–56.0)	17	45.0 (38.0–62.0)	0.76
Female (%)	33	52% (17)	17	71% (12)	0.20
DBD (%)	33	63% (21)	17	71% (12)	0.62
Height (cm)	33	175 (170–183)	17	170 (168–178)	0.25
TLC (L)	33	6.09 (5.43–7.50)	17	5.43 (5.30–6.82)	0.22
BMI	33	27 (24–30)	17	25 (23–28)	*0.07*
PaO_2_ at 100% (kPa)	33	35.4 (31.5–48.7)	17	54.9 (51.4–62.7)	**< 0.001**
Cause of death (%)	33		17		0.40
*Traumatic brain injury*		12% (4)		24% (4)	0.42
*Intracranial bleeding*		61% (20)		65% (11)	0.77
*Miscellaneous*		27% (9)		12% (2)	0.29

n (a), number available for analysis; M, medical EVLP; L, logistical EVLP; DBD, donation after brain death; TLC, total lung capacity; BMI, body mass index; PaO_2_, partial arterial oxygen pressure; kPa, kilopascal.

Data are presented as percentages (n) or as the median (IQR). Bold: significant value.

**Table 3:** Donor characteristics of conventional lung transplantation vs. lung transplantation after logistical EVLP (non-transplanted cases excluded).

	***n* (a)**	**Conventional LTx (*n* = 30)**	***n* (a)**	**LT (*n* = 15)**	***p*-value**
Age (years)	30	53.0 (35.0–63.3)	15	45.0 (37.0–58.0)	0.30
Female (%)	30	67% (20)	15	67% (10)	1.00
DBD (%)		63% (19)		67% (10)	0.83
Height (cm)	30	170 (165–179)	15	170 (168–180)	0.36
TLC (L)	30	5.43 (5.10–6.58)	15	5.43 (5.3–6.9)	0.40
BMI	30	25 (22–27)	15	26 (23–28)	0.20
PaO_2_ at 100% (kPa)	30	59.5 (52.8–64.0)	14	57.4 (51.6–63.1)	0.68
Cause of death (%)	30		15		0.46
*Traumatic brain injury*		13% (4)		27% (4)	0.41
*Intracranial bleeding*		60% (18)		60% (9)	1.00
*Miscellaneous*		27% (8)		13% (2)	0.24

n (a), number available for analysis; LTx, lung transplantation; LT, logistical EVLP with transplantation; DBD, donation after brain death; TLC, total lung capacity; BMI, body mass index; PaO_2_, partial arterial oxygen pressure; kPa, kilopascal.

Data are presented as percentages (n) or as the median (IQR).

The donor characteristics in the conventional LTx group did not differ significantly from the logistical EVLP cohort (Table 3).

### Recipient characteristics

Recipient characteristics are shown in Tables 4, 5. Recipients of LT lungs had a significantly lower most recent pre-transplantation FVC percentage (55% [46–74]) compared to the MT group (39% [28–46]; *p* = 0.01). LAS also differed significantly (33.5 [31.2–35.5] vs. 38.2 [35.0–41.2], respectively; *p* < 0.001) between these two groups (Table 4).

**Table 4:** Recipient and preservation characteristics of the medical EVLP and logistical EVLP groups.

	***n* (a)**	**MT (*n* = 26)**	***n* (a)**	**LT (*n* = 15)**	***p*-value**
Age (years)	26	59.5 (52.8–62.0)	15	59.0 (45.0–61.0)	0.27
Female (%)	26	46% (12)	15	73% (11)	*0.09*
BLTx (%)	26	96% (25)	15	100% (15)	1.00
CPB used (%)	26	12% (3)	15	0% (0)	0.29
ECLS used (%)	26	27% (7)	15	47% (7)	0.31
BMI	26	23 (20–27)	15	23 (19–27)	0.85
Last FEV1 %	26	24 (17–40)	15	23 (13–43)	0.46
Last FVC %	26	55 (46–74)	15	39 (28–46)	**0.01**
LAS	26	33.5 (31.2–35.5)	15	38.2 (35.0–41.2)	**< 0.001**
ICU stay (days)	26	5 (4–14)	15	6 (3–25)	0.86
Hospital stay (days)	23	29 (26–56)	14	43 (34–51)	0.12
Lung disease	26		15		0.51
*Emphysema*		62% (16)		40% (6)	0.18
*Cystic fibrosis*		15% (4)		13% (2)	1.00
*PPH*		0% (0)		7% (1)	0.37
*SPH*		4% (1)		7% (1)	1.00
*IPF*		12% (3)		27% (4)	0.39
*Miscellaneous*		8% (2)		7% (1)	1.00
Flush time (min)	20	14 (11–16)	9	12 (11–15)	0.23
1st CIT (min)	26	243 (222–272)	15	257 (232–302)	0.28
EVLP duration (min)	26	240 (229–286)	15	240 (180–292)	0.64
2nd CIT 1° lung (min)	25	264 (181–307)	15	237 (128–266)	0.10
2nd CIT 2° lung (min)	24	398 (310–438)	15	363 (268–398)	0.20
EVLP conversion rate	33	79% (26/33)	17	88% (15/17)	0.46

n (a), number available for analysis; MT, medical EVLP with transplantation; LT, logistical EVLP with transplantation; BLTx, bilateral lung transplantation; CPB, cardiopulmonary bypass; ECLS, extra-corporeal life support; BMI, body mass index; FEV_1_, forced expiratory volume in 1 s; FVC, forced vital capacity; LAS, lung allocation score; PPH, primary pulmonary hypertension; SPH, secondary pulmonary hypertension; IPF, idiopathic pulmonary fibrosis; CIT, cold ischemic time.

Data are presented as percentages (n) or as the median (IQR). Bold: significant value.

**Table 5:** Recipient and preservation characteristics of the conventional lung transplantation and lung transplantation after logistical EVLP groups.

	***n* (a)**	**Conventional LTx (*n* = 30)**	***n* (a)**	**LT (*n* = 15)**	***p*-value**
Age (years)	30	54.0 (37.5–60.3)	15	59.0 (45.0–61.0)	0.50
Female (%)	30	77% (23)	15	73% (11)	1.00
BLTx (%)	30	100% (30)	15	100% (15)	1.00
CPB used (%)	30	10% (3)	15	0% (0)	0.54
ECLS used (%)	30	23% (7)	15	47% (7)	0.17
BMI	30	22 (20–25)	15	23 (19–27)	0.48
Last FEV1 %	28	21 (15–33)	15	23 (13–43)	0.83
Last FVC %	28	49 (39–60)	15	39 (28–46)	0.11
LAS	30	36.9 (32.4–47.5)	15	38.2 (35.0–41.2)	0.27
ICU stay (days)	30	5 (3–11)	15	6 (3–25)	0.47
Hospital stay (days)	30	30 (22–39)	14	43 (34–51)	**0.008**
Lung disease	30		15		0.86
*Emphysema*		50% (15)		40% (6)	0.53
*Cystic fibrosis*		7% (2)		13% (2)	0.59
*PPH*		7% (2)		7% (1)	1.00
*SPH*		10% (3)		7% (1)	1.00
*IPF*		13% (4)		27% (4)	0.41
*Miscellaneous*		13% (4)		7% (1)	0.65
Flush time (min)		–	9	12 (11–15)	–
1st CIT (min)		–	15	257 (232–302)	–
EVLP duration (min)		–	15	240 (180–292)	–
2nd CIT 1° lung (min)		–	15	237 (128–266)	–
2nd CIT 2° lung (min)		–	15	363 (268–398)	–
CIT 1	29	338 (301–375)		–	–
CIT 2	29	457 (401–498)		–	–

n (a), number available for analysis; LTx, lung transplantation; LT, logistical EVLP with transplantation; BLTx, bilateral lung transplantation; CPB, cardiopulmonary bypass; ECLS, extra-corporeal life support; BMI, body mass index; FEV1, forced expiratory volume in 1 s; FVC, forced vital capacity; LAS, lung allocation score; PPH, primary pulmonary hypertension; SPH, secondary pulmonary hypertension; IPF, idiopathic pulmonary fibrosis; CIT, cold ischemic time.

Data are presented as percentages (n) or as the median (IQR). Bold: significant value.

Again, because of the matching procedure, recipient characteristics did not differ significantly at baseline between LT and LTx (Table 5).

The LT group had a significantly longer hospital stay than the conventional LTx group (43 [34–51] vs. 30 days [22–39], respectively; *p* = 0.008), but spent a similar length of time in ICU.

### Pulmonary graft function

#### Oxygenation

**Figure 1:**
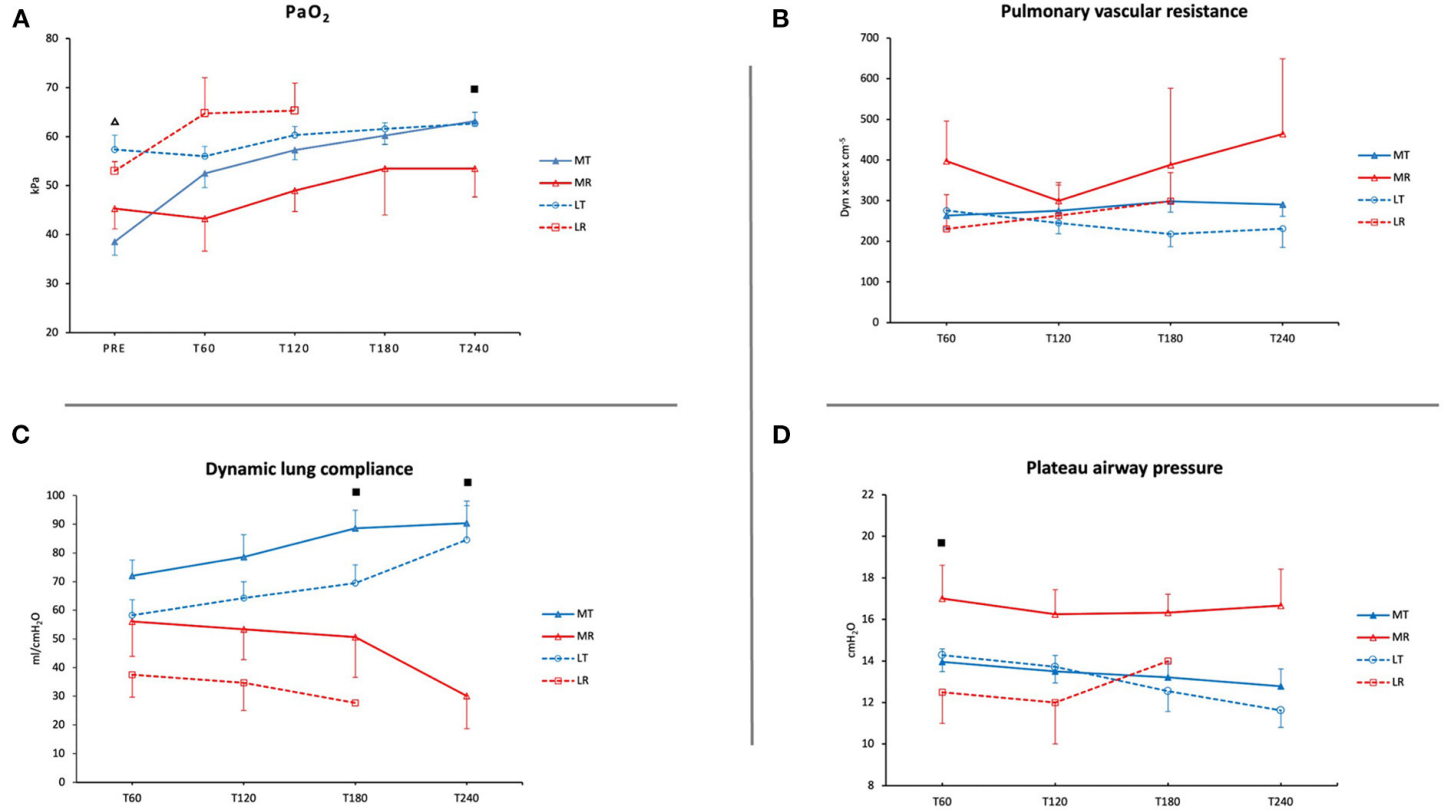
Pulmonary graft function during EVLP. **(A)** Oxygenation (PaO_2_ in kPa). **(B)** Pulmonary vascular resistance (PVR in dynes × s × cm^−5^). **(C)** Dynamic lung compliance (ml/cmH_2_O). **(D)** Plateau airway pressure (cmH_2_O). MT, medical EVLP with transplantation; MR, medical EVLP with rejection; LT, logistical EVLP with transplantation; LR, logistical EVLP with rejection. ^▵^MT vs. LT, *p* < 0.0001; ■MR vs. MT, *p* < 0.05.

During EVLP, there was an increase in PaO_2_ in MT, with a significant difference between pre-EVLP and T60, T120, T180, and T240 (38.56 ± 2.7 vs. 52.5 ± 2.9, 57.26 ± 1.9, 60.2 ± 1.8, and 63.2 ± 1.0 kPa; *p* < 0.001). Additionally, there was a significant difference in MT between T60 and T240 (52.5 ± 2.9 vs. 63.2 ± 1.0 kPa; *p* < 0.05). Pre-EVLP PaO_2_ was significantly lower in MT than in LT (38.56 ± 2.7 vs. 57.36 ± 2.9 kPa; *p* < 0.0001). PaO_2_ at T240 was lower in MR than in MT (53.47 ± 5.8 vs. 63.2 ± 1.0 kPa; *p* < 0.05) (Figure 1A).

#### Pulmonary vascular resistance

PVR increased in MR (*p* = NS) and LR during EVLP. In LT and MT, PVR decreased or remained stable (Figure 1B).

#### Dynamic lung compliance

Dynamic lung compliance increased during EVLP in LT (from 58.4 ± 5.4 to 84.6 ± 13.6 ml/cmH_2_O) and in MT (from 72 ± 5.6 to 90.5 ± 6.1 ml/cmH_2_O). However, it showed a decreasing trend in LR and MR from T120 until the end of the EVLP (*p* = NS in MR). There was a significant difference between MT and MR at T180 and T240 (88.6 ± 6.3 vs. 50.7 ± 14.1 ml/cmH_2_O and 90.5 ± 6.1 vs. 30.0 ± 11.2 ml/cmH_2_O; *p* < 0.05) (Figure 1C).

#### Plateau airway pressure

Plateau airway pressure (Pplat) was higher in MR and increased during EVLP in LR. At T60, Pplat was significantly lower in MT than in MR (13.9 ± 0.6 vs. 17.0 ± 1.6 cmH_2_O; *p* < 0.05) (Figure 1D).

### Primary graft dysfunction

**Figure 2:**
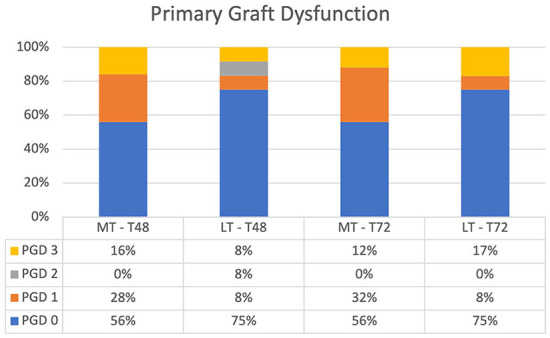
Primary graft dysfunction after lung transplantation at T48 and T72. There was no significant difference between MT and LT at T48 and T72. MT, medical EVLP with transplantation; LT, logistical EVLP with transplantation.

Overall, PGD grades between LT and MT showed no significant difference at T48 and T72 (Figure 2). PGD (grades 1–3) in LT was observed in 25% of the patients at T48 and T72. There was more grade 3 PGD at T72 than at T48 (17 vs. 8%). One patient with grade 3 PGD at T72 was on ECLS and one patient was weaned from ECLS at T24 but was still ventilated at T72.

In MT, 44% of patients had PGD (grades 1–3) at T48 and T72. At T72, ECLS was the reason for grade 3 PGD in two patients and prolonged ventilation with high oxygen demand and an edematous lung (identified by chest x-ray) in one patient.

In three patients in LT and in one patient in MT, ECLS was used for non-hypoxic reasons. Those patients were ungradable and excluded from the analysis.

Although not significantly different, there was more grade 0 PGD in LT than in MT at T48 and T72 (75 vs. 56%; *p* = NS).

**Figure 3:**
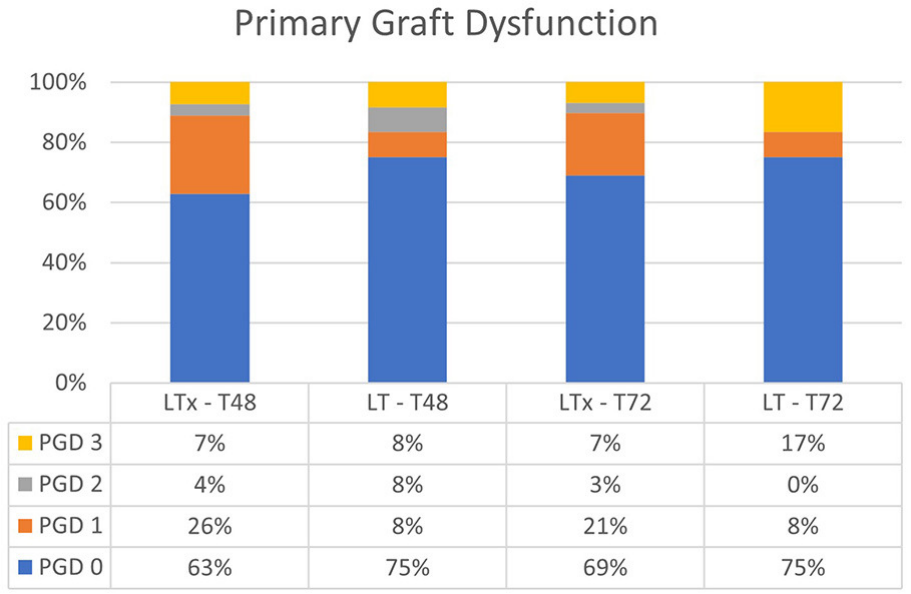
Primary graft dysfunction after lung transplantation at T48 and T72. There was no significant difference between LTx and LT at T48 and T72. LTx, standard lung transplantation; LT, logistical EVLP with transplantation.

When comparing the conventional LTx group with the LT group, overall PGD at T48 and T72 was not significantly different (*p* = 0.61 at T48, and *p* = 0.68 at T72; Figure 3). PGD (grades 1–3) in the LT group was observed in 25% of patients vs. 37% of patients in the conventional LTx group at T48. At T72, PGD (grades 1–3) was observed in 25 vs. 31% of patients, respectively. In the LT group, grade 3 PGD was observed in 8 and 17% of patients at T48 and T72, respectively. In the conventional LTx group, grade 3 PGD occurred in 7% of patients at both T48 and T72.

### Lung function

**Figure 4:**
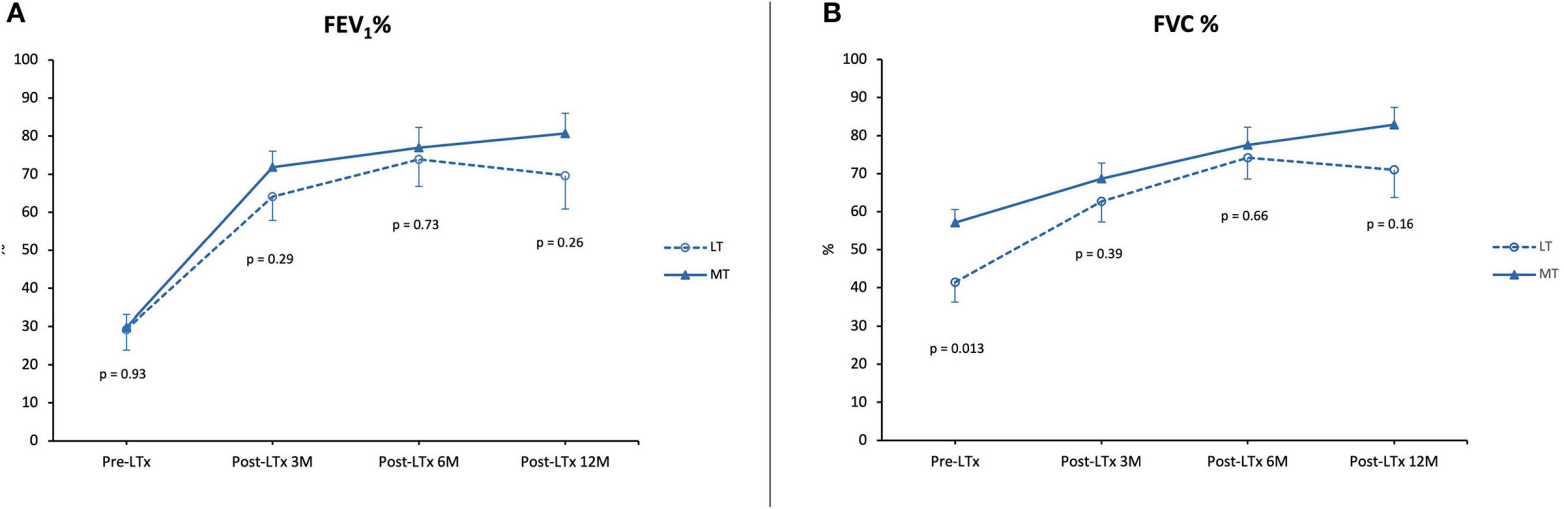
Lung function. **(A)** Forced expiratory volume in 1 second (FEV_1_) as a percentage of predicted pre-LTx and at 3, 6, and 12 months post-LTx. **(B)** Forced vital capacity (FVC) as a percentage of predicted pre-LTx and at 3, 6, and 12 months post-LTx. MT, medical EVLP with transplantation; LT, logistical EVLP with transplantation.

For LT vs. MT, FEV_1_ and FVC percentages are summarized in Figure 4. Data from the patient that underwent a single lung transplantation were excluded. Lung function after 1 year was comparable between MT and LT (FEV_1_ 80.7% ± 5.2 vs. 69.7% ± 8.8, *p* = NS; FVC 82.8% ± 4.6 vs. 71% ± 7.2, *p* = NS).

**Figure 5:**
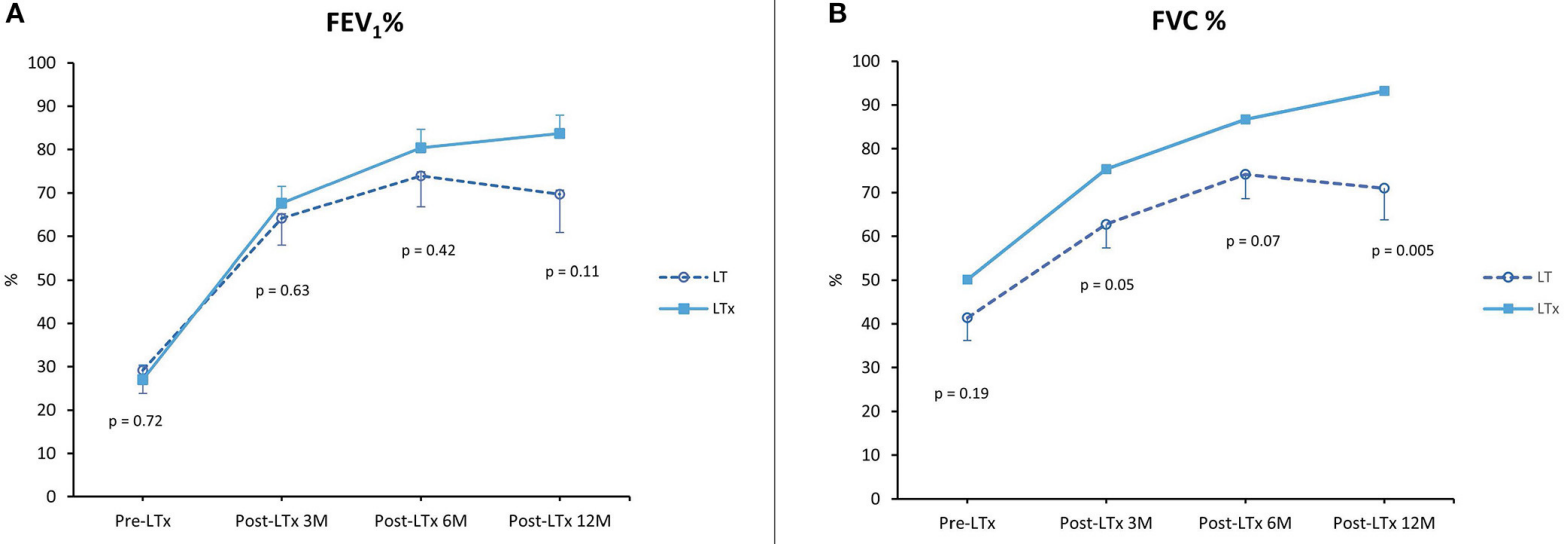
Lung function. **(A)** Forced expiratory volume in 1 second (FEV_1_) as a percentage of predicted pre-LTx and at 3, 6, and 12 months post-LTx. **(B)** Forced vital capacity (FVC) as a percentage of predicted pre-LTx and at 3, 6, and 12 months post-LTx. LTx, standard lung transplantation; LT, logistical EVLP with transplantation.

In the LT vs. LTx group, FEV_1_ and FVC percentages are summarized in Figure 5. FEV_1_ percentage 1 year after transplantation was comparable between LT and LTx (FEV_1_ 69.7% ± 8.8 vs. 83.8% ± 4.2; *p* = NS). By contrast, FVC percentage in the LT group was significantly lower than in the LTx group (71.0% ± 7.2 vs. 93.3% ± 3.8; *p* = 0.005).

### Chronic lung allograft dysfunction

**Figure 6:**
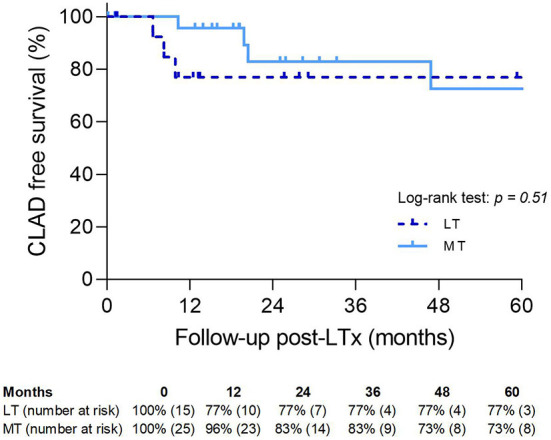
Chronic lung allograft dysfunction-free survival. MT, medical EVLP with transplantation; LT, logistical EVLP with transplantation.

Five-year CLAD-free survival was slightly better in LT than in MT (77 vs. 73%; *p* = NS) (Figure 6). One patient died 3 days after a single lung transplantation (re-transplantation) and was excluded from the analysis.

**Figure 7:**
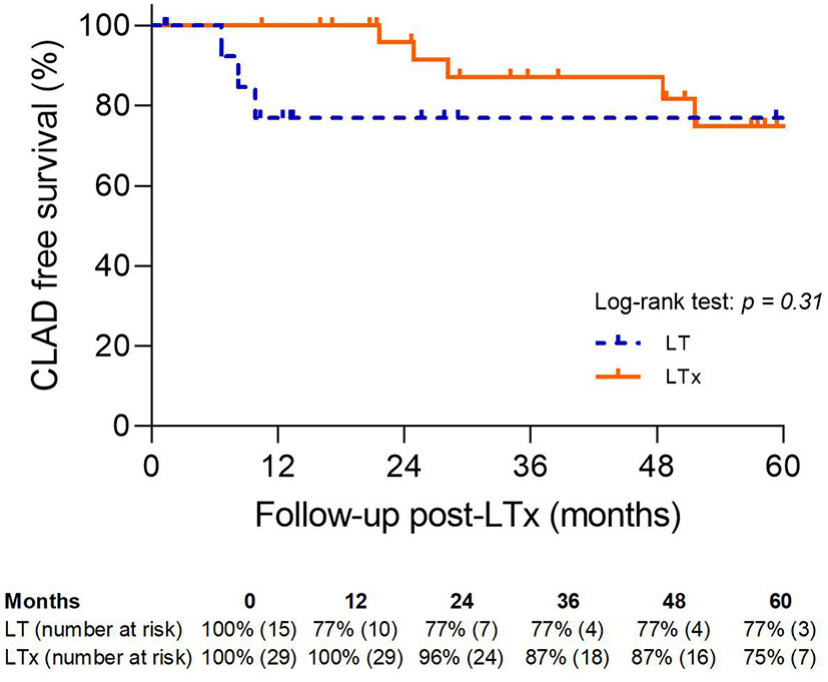
Chronic lung allograft dysfunction-free survival. LTx, standard lung transplantation; LT, logistical EVLP with transplantation.

There was no significant difference in 5-year CLAD-free survival between the LT and LTx groups (77 vs. 75%; *p* = NS) (Figure 7).

### Survival

**Figure 8:**
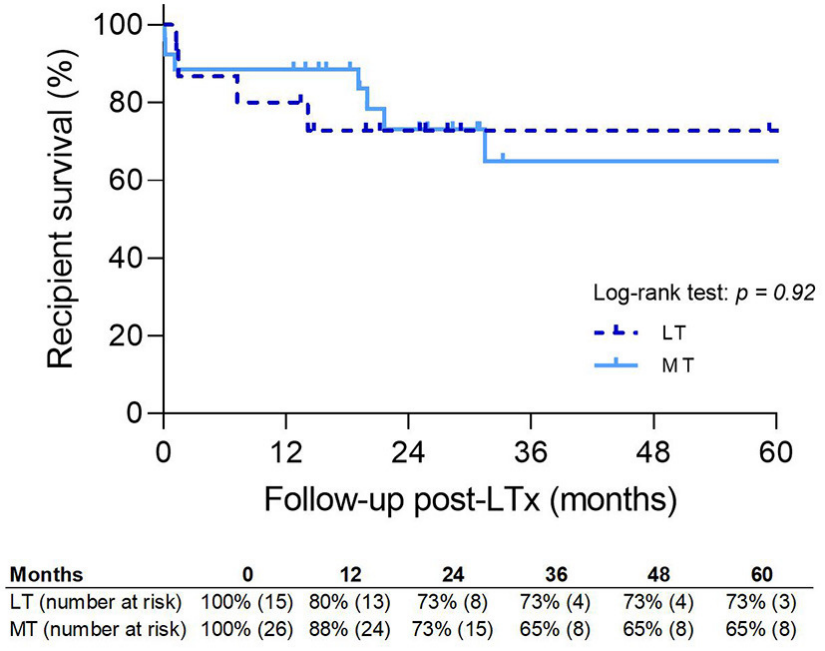
Patient survival. MT, medical EVLP with transplantation; LT, logistical EVLP with transplantation.

Recipient survival is shown in Figure 8. There was no significant difference between LT and MT; however, 5-year survival was slightly better in LT. Seven patients in the MT group died during follow-up. Two patients died within 30 days of either a massive pulmonary embolism (*n* = 1) or intracranial infarction (*n* = 1), and one patient died of haemorrhagic shock within 90 days. CLAD was the cause of death in two patients (19 and 21 months after transplantation). One patient died of a metastasized lung carcinoma and one patient died because of an intracranial bleed.

In the LT group, four patients died during follow-up. Within 90 days, two patients died of either hemoptysis due to a fistula from the right pulmonary artery to the right bronchus (*n* = 1) or of circulatory insufficiency due to multiple thrombi in the great vessels, which did not respond to treatment (*n* = 1). CLAD was the reason for death in one patient 25 months post-transplant, and myeloencephelopathy due to JC virus caused the death of another patient.

**Figure 9:**
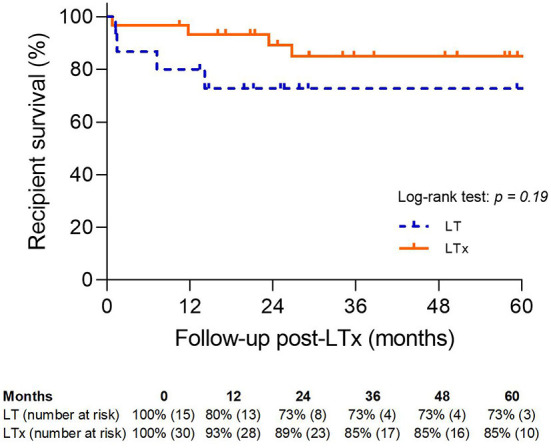
Patient survival. LT, logistical EVLP with transplantation; LTx, standard lung transplantation.

Recipient survival was comparable between the LT and LTx groups (73 vs. 85%; *p* = NS) (Figure 9).

## Discussion

In this study, we showed that logistically motivated EVLP is safe and feasible. Post-transplant, both groups performed similarly, although there was a tendency for longer CLAD-free survival and better overall survival in the logistically motivated EVLP group. When comparing the LT group with a conventional group, we found no significant differences in CLAD-free survival and overall survival.

A secondary outcome was that we confirmed that compliance and airway pressures in our protocol are more valuable than oxygenation capacity when assessing lung quality.

Furthermore, in the LR group, we demonstrated reduced pulmonary graft function after 2 h of EVLP, which likely prevented the transplantation of lungs of unidentified inferior quality. Finally, and importantly, we also found that the LT group had numerically less PGD than the MT group, and that PGD was also numerically less in LT than in LTx.

The good functional result observed after logistical EVLP was expected based on the experience of other studies, particularly that of the Toronto group. Yeung et al. observed that a total preservation time of more than 12 h was not detrimental to the development of PGD. At 72 h after transplantation, grade 3 PGD was 10%. Additionally, a longer preservation time did not influence survival after lung transplantation, with a 1-year mortality of 13% (6). This observation is in line with another article by the Toronto group in which grade 3 PGD at 72 h in the logistics group was 0%, with a 30-day mortality of 0% (13). In our study, both groups had a prolonged preservation time. In the LT group, our aim was to bridge the night-time period, electively prolonging preservation time and enabling the timing of transplantation. In one case, it also enabled us to perform a sequential heart and lung transplantation from the same donor. The higher grade-3 PGD in the LT group was caused by the higher complexity of these transplantations, which require prolonged ECLS. In MT, grade 3 PGD was comparable with LT, with ECLS still necessary for two patients. In addition to the good functional outcome of LT EVLP, it should be emphasized that control of timing is motivating for the whole team and raises the transplantation of difficult recipient cases to a higher level. Although not measurable in our study, it is conceivable that the former contributes to a better overall outcome.

The desire for daytime lung transplantation surgery is hampered by the fact that, for many medical centers, it increases costs (14). However, for our institution, which is a referral cardiothoracic center, prevention of night-time lung transplantation allows us more flexibility when arranging adequate cardiothoracic emergency care. Therefore, we now train institutional organ perfusion personnel for all donor organs, including EVLP. This is supported by the fact that, in the Netherlands, EVLP has been included in the health insurance budget. The use of EVLP allowed us to increase our preservation time from 8 h up to 14 h, with no negative effects on early graft function. The Toronto group demonstrated that preservation can be safely extended for at least 12 h, even for high-risk donors (6). This facilitates a situation in which several organs can be accepted at the same time and difficult recipient cases can be managed.

When analyzing donor lung quality, oxygenation capacity was long seen as the most important parameter for assessing lungs before and during EVLP. There is increasing evidence that overall graft performance during EVLP is more important for evaluation (7, 13, 15, 16). A study by Yeung et al., in which an acellular perfusate was used, demonstrated that while compliance and airway pressure deteriorated, oxygenation remained stable. However, Okamoto et al. described a correlation between the P/F ratio and the airway parameters. In this study, a cellular perfusate was used and the Lund protocol was followed.

Additionally, oxygenation may be stable after 2 h of EVLP, with no significant improvement afterwards (17). The necessity for a holistic view during EVLP is supported by our current finding that there was no significant difference in oxygenation capacity between rejected and transplanted pulmonary grafts. We found that a decrease in compliance and high or increased airway pressure after 2 h of EVLP were the decisive parameters when rejecting lungs, even when they initially met the acceptance criteria for transplantation. This was also demonstrated in our previous animal study of *in situ* lung perfusion, in which compliance and plateau airway pressures were better in less badly injured donor lungs (18). Two lungs with a pO_2_ above 60 kPa were declined for transplantation due to a decrease in compliance, an increase in plateau airway pressure and pulmonary vascular resistance, and an obvious pulmonary edema upon inspection. This was also observed in a prospective study in which standard donor lungs were subjected to EVLP, suggesting that these lungs can deteriorate during EVLP (8). Di Nardo et al. (19) outlined the cutoff values for EVLP parameters that, in the near future, may help centers to better interpretate the parameters for rejection or acceptance of lungs. They describe the variation over time for ventilatory parameters, lung metabolism, and gas exchange. The use of dynamic lung compliance/total lung capacity and static lung compliance/total lung capacity of the donor are recommended for generalizing the data. When comparing the proposed cutoff values with our data from the LT group, compliance was acceptable at T60 in the first case but not at T120. In the second case, compliance was already lower than the cutoff value at the first evaluation.

A protective ventilation strategy during EVLP is one of the cornerstones of a successful procedure. Lungs are ventilated with a tidal volume of 7 ml/kg, and during the evaluation phase this is increased up to 10 ml/kg. This mechanical ventilation without support from the chest wall, mediastinum, and diaphragm may lead to overdistention of the lung, high end-inspiratory lung volumes, and high lung strain, and can result in alveolar damage and subsequent inflammatory responses. This ventilator induced lung injury might lead to capillary leakage and pulmonary edema (20). Also, atelectrauma at the start of the ventilation plays an important role. In retrospect, we hypothesize that the deterioration of the two rejected LT lungs during EVLP might be influenced by our initial larger tidal volume ventilation during the 10 min of assessment measurements.

During the ventilation of lungs in a supine position, there is more atelectasis and shunting in the dependent parts compared with ventilation in a prone position. The supine position also causes reduced blood flow in non-dependent lung regions (21). Persistent atelectasis or low oxygenation capacity prompted the decision to change lungs from a supine position to a prone position during our EVLPs. This resulted in improved recruitment of the alveoli and redistribution of blood flow, with better oxygenation. The prone position may also influence the clearance of edema fluid as alveolar epithelial function is improved due to better ventilation of the non-dependent alveoli. Niikawa et al. (22) demonstrated in a case report that placement of lungs in a prone position immediately after the start of the EVLP followed by a change to a supine position during the last hour of EVLP reduced pulmonary edema in one of the two cases. In a randomized study, using lungs that were declined for transplantation, EVLP in a prone position was compared with EVLP in a supine position. After 2 h of EVLP, P/F ratio and lung weight improved in three out of five lungs in the prone group, whereas none of the lungs in the supine group were deemed suitable for transplantation (23).

In a previous study, our first experience with medical EVLP was compared with a non-EVLP group (N-EVLP) (10). The EVLPs performed for logistical reasons were excluded from this study. The 1-year survival in N-EVLP was 89%, compared with 80% in LT. There was one patient diagnosed with CLAD at 24 months post-LTx in N-EVLP, and one patient with CLAD at 25 months in LT. In N-EVLP, PGD (grades 1–3) was observed in 22% of patients at T48 and in 28% of patients at T72 vs. PGD (grades 1–3) in 25% of patients at T48 and T72 in LT.

In the present study, we compared the LT group with a conventional LTx group. We observed no significant differences between the two groups for PGD, CLAD-free survival, and recipient survival. However, PGD (grades 1–3) was slightly lower at T48 and T72 in LT. Several meta-analyses comparing lung transplantation after EVLP with lung transplantation after standard cold storage have reported a lower incidence of PGD after EVLP and a similar post-operative outcome in both groups (5, 24). When lungs that met the standard criteria for lung transplantation were subjected to EVLP, outcomes were comparable and a lower incidence of grade >1 PGD was reported in the EVLP group. However, in this study, patients with pulmonary arterial hypertension and severe risk factors were excluded (8).

Extensive lung injury caused by PGD may increase the risk of CLAD development (25). In a previous study, we did not observe a correlation with CLAD development (10). This was confirmed by Divithotawela et al. (26) who did not observe a difference in CLAD-free survival between the EVLP and non-EVLP group. In our study, two patients with grade 3 PGD, one in MT and one in LT, developed CLAD in the follow-up. However, there was no correlation between CLAD and PGD in MT or LT.

In our study, the conversion rate was 79% in MT and 88% in LT. However, in two cases during EVLP for medical reasons, one lung of the lung block was suitable for transplantation. The single lung allograft was offered for re-allocation. Unfortunately, there was no suitable recipient. This could have increased our conversion rate for medical EVLP to 85%. Divithotawela et al. (26) described more single lung transplants in the EVLP group due the unique possibility of salvaging one lung during EVLP rather than discarding both lungs.

Two organs were subjected to EVLP because of thrombi in the pulmonary artery. In one case, the whole right pulmonary artery was filled with thrombus. This was mechanically removed but, in the future, EVLP should enable the implementation of protocols for administering fibrinolytics during organ preservation, thereby improving organ function. The use of alteplase and urokinase for lung reconditioning during EVLP, followed by successful transplantation, was mentioned in two case reports (27, 28). In 2013, Machuca et al. reported the use of 20 mg of alteplase during EVLP. Inci et al. demonstrated that the administration of 100,000 IU of urokinase dissolved the microthrombi, resulting in an improved P/F ratio, pulmonary vascular resistance, and compliance.

We acknowledge the limitations of our study. First, we adapted the ventilation protocol because of the risk of developing ventilator-induced lung injury.

Second, in the LT group, two sets of lungs were rejected for transplantation. We hypothesize that this was caused by unrecognized allograft dysfunction; however, ventilator-induced lung injury should not be overlooked as the cause of the pulmonary edema. Therefore, further evaluation of ventilation strategies is necessary. Third, the LT group was only a small group. As PGD is also dependent on the recipient and the recipient operation, the relationship between the LT group and PGD should be the subject of further studies.

Fourth, the study was a retrospective comparison. We began with MT EVLP, but LT EVLP was started later, which means that improved experience might contribute to the excellent results observed in LT.

In conclusion, the transplantation of lungs treated with EVLP for logistic reasons is possible. This method makes the transplantation of lungs with otherwise very extended cold ischemia times more controllable, and complicated lung transplantations during the daytime possible. Moreover, EVLP for logistical reasons leads to outcomes that are comparable with conventional lung transplantation.

Furthermore, this study confirms that EVLP is an excellent tool for reconditioning injured lungs. An important finding of this study is that in the function evaluation on EVLP lung compliance and plateau airway pressure are proved more valuable than PaO_2_.

## Data availability statement

The raw data supporting the conclusions of this article will be made available by the authors, without undue reservation.

## Ethics statement

The studies involving human participants were reviewed and approved by METc 2021/339, UMCG research register number: 202100305. Written informed consent for participation was not required for this study in accordance with the national legislation and the institutional requirements.

## Author contributions

CV, VS, ZZ, and RU collected and analyzed the data. CV, VS, ZZ, EV, WB, CG, RU, and ME wrote the manuscript. All authors contributed to the article and approved the submitted version.
